# Superior immune responses induced by intranasal immunization with recombinant adenovirus-based vaccine expressing full-length Spike protein of Middle East respiratory syndrome coronavirus

**DOI:** 10.1371/journal.pone.0220196

**Published:** 2019-07-22

**Authors:** Myung Hee Kim, Hyun Jik Kim, Jun Chang

**Affiliations:** 1 Graduate School of Pharmaceutical Sciences, Ewha Woman’s University, Seoul, Republic of Korea; 2 Department of Otorhinolaryngology, Seoul National University College of Medicine, Seoul, Republic of Korea; Instituto Butantan, BRAZIL

## Abstract

Middle East respiratory syndrome coronavirus (MERS-CoV) causes an acute and severe lower respiratory illness as well as vomiting, diarrhea, and renal failure. Because no licensed MERS-CoV vaccines are currently available, preventive and therapeutic measures are urgently needed. The surface spike (S) glycoprotein of MERS-CoV, which binds to the cellular receptor dipeptidyl peptidase 4 (DPP4), is considered as a major target for MERS-CoV vaccine development. Here, we designed recombinant replication-deficient adenovirus-based vaccines expressing the N-terminal domain (rAd/NTD) and receptor-binding domain (rAd/RBD) of the MERS-CoV S1 subunit and full-length Spike protein (rAd/Spike). We found that immunization with candidate vaccines via intranasal route induced S1-specific IgG antibodies and neutralizing antibodies against MERS spike pseudotyped virus. Especially, rAd/Spike induced the highest neutralizing antibody titer and the strongest cytokine-induced T cell responses among the three candidate vaccines. To compare the immune responses induced by different administration routes, rAd/Spike was administered via intranasal, sublingual, or intramuscular route. All these administration routes exhibited neutralizing effects in the serum. MERS-CoV-specific neutralizing IgA antibodies in the bronchoalveolar lavage fluid were only induced by intranasal and sublingual administration but not by intramuscular administration. Intranasal administration with rAd/Spike also created resident memory CD8 T cells in the airway and lung parenchyma. Taken together, our results showed that both the humoral and cellular immune responses are highly induced by rAd/Spike administration, suggesting that rAd/Spike may confer protection against MERS-CoV infection.

## Introduction

Middle East respiratory syndrome coronavirus (MERS-CoV) has emerged as a novel lethal human pathogen which causes severe acute respiratory infection since its identification in Saudi Arabia in 2012 [1]. The virus causes respiratory diseases such as fever, cough, and shortness of breath, which may lead to pneumonia and acute renal failure in immunocompromised patients and the elderly [1–7]. To date, 2,266 laboratory-confirmed MERS-CoV infection cases including 804 related deaths from 27 countries have been reported to World Health Organization (http://www.who.int/emergencies/mers-cov/en/). Currently, such cases are increasingly being reported. MERS-CoV also has the potential for a widespread outbreak, as observed in 2015 in South Korea [8]. Currently, the lack of prophylactic or therapeutic measures has created an urgent need for effective vaccine the development against MERS-CoV infections [9,10].

MERS-CoV is an enveloped positive-sense single-stranded RNA virus that belongs to lineage C of the genus *Betacoronavirus* (β-CoV) in the family *Coronaviridae* [11–13]. The MERS-CoV genome encodes four structural proteins: spike (S), envelope (E), membrane (M), and nucleocapsid (N) [14,15]. The S protein, a type I transmembrane glycoprotein on the virus surface, contains two functional subunits and mediates viral infection. The S1 subunit comprises N-terminal domain (NTD) and receptor-binding domain (RBD) that recognizes and binds to dipeptidyl peptidase 4 (DPP4, also known as CD26) receptor on the host cell surface to initiate infection [16–18]. The S2 subunit with two heptad repeats (HR), HR1 and HR2, facilitates fusion between the virus and host cell membrane [19,20]. The S glycoprotein is considered as a suitable vaccine candidate for eliciting neutralizing antibodies to block viral entry or neutralize viral infection [21–23].

The Spike protein-based vaccine candidates have been developed and investigated, such as whole-inactivated virus [24], subunit vaccines [22,25–29], DNA-based vaccines [27,30,31], and viral vector-based vaccines [32–36], using both the Spike protein as a whole and Spike protein fragments. These vaccine candidates induced humoral immune responses and neutralizing antibody responses as well as cellular immune responses. However, a vaccination strategy focused on RBD alone has several issues that may not be optimal for generating neutralizing antibody responses [27] and can only induce partial protection against the MERS-CoV infection [29,37]. Detailed structural analysis revealed that RBD-specific antibodies stimulate the emergence of viral escape mutation in this region; therefore, the generation of more diverse antibodies directed toward both RBD and outside the domain may be a better strategy for developing effective vaccines [27,38].

Here, we designed and constructed recombinant adenovirus-based vaccine candidates encoding NTD, RBD, and full-length Spike protein. We assessed their immunogenicity and compared immune responses generated from vaccination via different routes and found that rAd/Spike could induce the strongest humoral and cellular immune responses. Additionally, immunization with rAd/Spike conferred long-lasting neutralizing antibody responses, suggesting that rAd/Spike is the best candidate for an effective MERS-CoV vaccine development.

## Materials and methods

### Mice and ethics statement

Female BALB/c mice aged 6 weeks were purchased from Orient Bio Inc. (Seoul, Korea). All mice were maintained under specific pathogen-free conditions in the experimental facility at Ewha Woman’s University. All mice were group housed in cages, containing four mice each, at approximately 22°C with 12 h light/dark cycle. Cage changes were performed once weekly until the end of the experiment, and food and water were replenished as needed. During the study, mice were checked daily to ensure health and animal welfare. All mice were sacrificed by CO_2_ euthanasia. All animal studies were approved by Ewha Woman’s University Institutional Animal Care and Use Committee (IACUC; Approval No. 18–005) and carried out under strict compliance with the suggestions given by the Institute of Laboratory Animal Resources Guide for the Care and Use of Laboratory Animals.

### Cell culture

HEK293 cells (ATCC CRL-1573) were cultured in MEM (Welgene, Korea) containing 10% fetal bovine serum (FBS). 293TN cells and Huh7.5 cells were purchased from ATCC collection (Manassas, VA) and were grown in DMEM (Welgene) supplemented with 10% FBS. *Spodoptera frugiperda* 21 (Sf-21) insect cells (Invitrogen, Carlsbad, CA, USA) were propagated in SF-900 II serum-free medium (Gibco BRL) at 28°C and used to produce a recombinant NTD (rNTD) protein.

Normal human nasal epithelial (NHNE) cells were cultured as described previously [39]. Briefly, passage-2 NHNE cells (1×10^5^ cells/culture) were seeded in 0.25 ml of culture medium on Transwell clear culture inserts (24.5-mm, with a 0.45-mm pore size; Costar Co., Cambridge, MA, USA). Cells were cultured in a 1:1 mixture of basal epithelial growth medium and DMEM containing previously described supplements. Cultures were grown while submerged for the first 9 days. The culture medium was changed on day 1, and every other day thereafter. An air–liquid interface (ALI) was created on day 9 by removing the apical medium and feeding the cultures from the basal compartment only. The culture medium was changed daily after the initiation of the ALI. We add antibiotics such as 1% penicillin and streptomycin into the all media for subculture and culture stages and we also add antifungal agent, fungizone (1 ml/1,000 ml media) (Life technologies, Grand island, NY, USA) after filtering the media. All experiments described here used cultured nasal epithelial cells at 14 days after the creation of the ALI.

### Recombinant replication-defective adenovirus construction

The amino acid sequence of the MERS-CoV Spike protein was from MERS-CoV/KOR/KNIH/002_05_2015 (GenBank KT029139, residues 1–1353). Spike gene was codon-optimized and synthesized for mammalian cell expression (GenScript, Piscataway, NJ). An optimal Kozak translation sequence was also included immediately upstream of ATG. This synthetic DNA was subcloned into a pShuttle-CMV vector through *Bgl*II/*Not*I double digestion and used as templates for polymerase chain reaction (PCR) amplification. Subsequently, constructs expressing different MERS-CoV Spike protein fragments were designed. The coding sequence for RBD (residues 367–606) was amplified by PCR using the forward (5ʹ-GA**GCTAGC**GAGGCCAAGCCCTCTGGC-3ʹ) and reverse primers (5ʹ-GA**CTCGAG**TTATCACTTGTCATCATCGTCCTTGTAGTCGTACTCCACGCAATTGCCCAG-3ʹ), which contain two stop codons and a FLAG tag sequence. The coding sequence for NTD (residues 18–353) was amplified by PCR using the forward (5ʹ-GA **GCTAGC**TACGTCGATGTGGGACC-3ʹ) and reverse primers (5ʹ-GA**CTCGAG**TTATCAGTGGTGGTGGTGGTGATGGGACTCATAGCTACAGTGCAG-3ʹ) which contain two stop codons and a six-histidine tag sequence. Finally, the PCR products of RBD and NTD gene were inserted into the pShuttle-CMV vector through *Nhe*I/*Xho*I double digestion, respectively. The pShuttle-CMV plasmid was designed to include the start codon and the signal sequence of human tissue plasminogen activator (tPA) upstream of the transgene sequence. Replication-defective adenoviruses (serotype 5) were generated by inserting foreign sequences via homologous recombination and subsequent purification of recombinant adenovirus (rAd), as described previously [40]. Briefly, the shuttle vector plasmid was electroporated into electrocompetent BJ5183 cells carrying the pAdEasy-1 adenoviral genomic DNA. Recombinant adenoviral DNA was isolated and linearized with *Pac*I and subsequently transfected into HEK293 cells to generate rAd/Spike, rAd/RBD, and rAd/NTD viruses. Mock adenovirus (rAd/mock) was generated by the same method using the empty pShuttle-CMV vector. The recombinant viruses were amplified on HEK293 cells and purified by double-cesium chloride (CsCl_2_) gradient ultracentrifugation. The expression and secretion of Spike and RBD proteins by rAd-infected HEK293 cells were verified via Western blot analysis by employing anti-MERS-CoV S1 protein rabbit polyclonal antibody [amino acids (aa) 1–725; 40069-T48; Sino Biological Inc., Beijing, China] and HRP-conjugated goat anti-rabbit IgG antibody (Abcam, Cambridge, UK). The expression and secretion of NTD protein were validated by infecting HEK293 cells and by conducting Western blot analysis using rAd/Spike-immunized mouse serum (1:1,000 dilution) and HRP-conjugated rabbit anti-mouse IgG antibody (Abcam) as a secondary antibody.

### Mouse immunizations

Female BALB/c mice aged 6–8 weeks were immunized with rAd vaccine or control virus via intranasal (IN), intramuscular (IM), and sublingual (SL) routes. For IN immunization, mice were lightly anesthetized via isoflurane (Ifran; Hana Pharm., Kyonggi-Do, Korea) inhalation, and 50 μl of vaccine or control virus solution was inoculated into the left nostril. For IM immunization, 100 μl of the vaccine was injected on the right hind leg of mice. For SL immunization, mice were anesthetized with an intraperitoneal (i.p.) injection of 100 mg/kg body weight ketamine (Yuhan Co., Seoul, Korea) and 10 mg/kg body weight Rompun (Bayer, Seoul, Korea) mixture in PBS, and subsequently, 15 μl vaccine was inoculated under their tongue. Blood was collected from the retro-orbital plexus using a heparinized capillary tube; further, blood samples were centrifuged and sera were obtained and stored at −70°C. Mice were sacrificed at 1, 2, and 19 weeks after undergoing the last immunization, and tracheotomy was performed. Bronchoalveolar lavage fluid (BALF) was collected by washing the lung airways with 1 ml PBS. BALF was centrifuged and the supernatant was used to measure secretory IgA and neutralizing antibody titers.

### Expression and purification of recombinant NTD protein

The rNTD protein was prepared using a Bac-to-Bac baculovirus expression system (Invitrogen) according to the manufacturer’s protocol. The NTD gene amplified by PCR, as described above, was cloned into the pFastBac1 vector with a 5ʹ-terminal tPA for protein secretion. This expression cassette was inserted into the baculovirus genome within DH10Bac (Invitrogen). The recombinant bacmid-encoding NTD (rBac-NTD) plasmid was transfected into Sf-21 cells using Cellfectin transfection reagent (Invitrogen). The primary recombinant baculovirus was harvested 5 days after transfection and then amplified by infecting into new large-scale Sf-21 cell cultures. To obtain the rNTD protein, final culture supernatants of infected Sf-21 cells were collected and loaded onto a 5-ml HisTrap HP column (GE Healthcare) with a binding buffer (20 mM KPO_4_, 500 mM NaCl, 10 mM imidazole, pH 7.4). The column was washed with binding buffer and eluted using an elution buffer (20 mM KPO_4_, 500 mM NaCl, 500 mM imidazole, pH 7.4). After removing most impurities, the eluted target protein fractions were pooled, concentrated, and buffer-exchanged with PBS. Purified rNTD protein concentration was determined using the NanoDrop 2000 Spectrophotometer (Thermo Fisher Scientific).

### ELISA

The antigen-specific IgG and IgA antibody titers in immunized mice were determined by a direct enzyme-linked immunosorbent assay (ELISA). Briefly, 96-well plates (Nunc MaxiSorp; Thermo Fisher Scientific) were coated with 200 ng/well of S1 (aa 1–725; 40069-V08B1), S2 (aa 726–1296; 40070-V08B), RBD (aa 367–606; 40071-V08B1) (Sino Biological Inc.), or rNTD (aa 18–353) of MERS-CoV Spike protein in 100 μl PBS and incubated overnight at 4°C. Each antigen-coated well was blocked with PBS containing 1% non-fat milk and 0.05% Tween 20 for 2 h at room temperature (RT). Subsequently, serial dilutions of sera or BALFs were added into the well and incubated for 2 h at RT. After washing with PBS containing 0.05% Tween 20, HRP-conjugated rabbit anti-mouse IgG (Abcam) or HRP-conjugated goat anti-mouse IgA (Zymed Laboratories, San Francisco, CA) was added as a secondary antibody and incubated for 1 h at RT under dark conditions. The plates were washed, and the reaction was developed with 3,3ʹ,5,5ʹ-tetramethylbenzidine (TMB) peroxidase substrate (KPL, Gaithersburg, MD); gradually, the reaction was stopped with 1 M H_3_PO_4_ and analyzed at 450 nm wavelength using Thermo Multiskan EX (Vantaa, Finland).

### MERS-CoV Spike pseudotyped lentivirus production

The GFP-expressing lentiviral transfer plasmid was constructed using pLenti6 Gateway Vector kits (Invitrogen) according to the manufacturer’s protocol. The full-length MERS-CoV spike protein-expressing envelope plasmid was constructed by inserting a codon-optimized synthetic DNA sequence (GenScript) into pCAGG plasmid (Addgene, www.addgene.org). To produce MERS-CoV Spike pseudotyped lentivirus, 293TN cells were co-transfected with packaging plasmids pLP1 and pLP2, the lentiviral transfer plasmid, and the envelope plasmid pCAGG/MERS-CoV Spike using Lipofectamine 2000 transfection regent (Invitrogen). After overnight incubation, the transfection medium was replaced with fresh 10% DMEM. Forty-eight hours later, culture supernatants containing pseudovirus were collected, centrifuged to remove debris, passed through 0.45-μm filter, and stored at −70°C.

### Pseudovirus neutralization assay

Huh 7.5 cells (30,000 cells per well) were plated in 48-well plates on the day before neutralization assay. Sera and BALFs were heat-inactivated at 56°C for 30 min. MERS-CoV Spike pseudovirus was pre-incubated with serially diluted sera or BALFs for 1 h at 37°C, and the mixture was added to Huh 7.5 cells. After overnight incubation, cells were changed with fresh 10% DMEM and cultured for another 48 h. Subsequently, the cells were harvested with trypsin-EDTA and analyzed using FACSCalibur flow cytometer (BD Biosciences, San Diego, CA). MERS-CoV pseudovirus transduction was measured as % transduction.

### Real-time PCR for MERS-CoV

NHNE cells were infected with virus solution (MERS-CoV 10 μl/PBS 10 ml) (Korea Centers for Disease Control and Prevention, Korea) for 1 day and total RNA was isolated using TRIzol (Life technology, Seoul, Korea). cDNA was synthesized from 3 μg of RNA with random hexamer primers and Moloney murine leukemia virus reverse transcriptase (Perkin Elmer Life Sciences, Waltham, MA, USA and Roche Applied Science, Indianapolis, IN, USA). Amplification was performed using the TaqMan Universal PCR Master Mix (PE Biosystems, Foster City, CA, USA) according to the manufacturer’s protocol. Briefly, amplification reactions had a total volume of 12 μl and contained 2 μl of cDNA (reverse transcription mixture), oligonucleotide primers (final concentration of 800 nM), and TaqMan hybridization probe (200 nM). Real-time PCR probes were labeled at the 5’ end with carboxyfluorescein (FAM) and at the 3’ end with the quencher carboxytetramethylrhodamine (TAMRA). To quantify the cellular viral level and host gene expression, cellular RNA was used to generate cDNA. The MERS-CoV mRNA level was monitored using a quantitative PCR for the *N2* gene of MERS. Primers for *N2* gene of MERS-CoV was purchased from Applied Biosystems (Foster City, CA, USA). Real-time PCR was performed using the PE Biosystems ABI PRISM 7700 Sequence Detection System. Thermocyling parameters were as follows: 50°C for 2 min, 95°C for 10 min, and then 40 cycles of 95°C for 15 s and 60°C for 1 min. All PCR assays were quantitative and utilized plasmids containing the target gene sequences as standards. All reactions were performed in triplicate, and all real-time PCR data were normalized to the level of the housekeeping gene glyceraldehyde phosphate dehydrogenase (GAPDH, 1×10^6^ copies) to correct for variations between samples.

### Flow cytometric analysis

Using a syringe, the lungs were perfused with 5 ml PBS containing 10 U/ml heparin (Sigma, St. Louis, MO) via the right ventricle. To obtain single-cell suspensions, the tissues were homogenized and passed through 70-μm cell strainers (SPL, Kyonggi-Do, Korea). For MHC class I tetramer analysis, lymphocytes were washed with FACS buffer (0.5% FBS and 0.1% NaN3 in PBS) and were blocked with purified rat anti-mouse CD16/CD32 (BD Pharmingen, San Diego, CA) and 5 μg/ml streptavidin (Invitrogen). Then, cells were stained with anti-CD8a [53–6.7], anti-CD44 [IM7] (BioLegend, San Diego, CA), and K^d^/S291 (KYYSIIPHSI) tetramer for 30 min at 4°C under dark conditions. After staining, the cells were fixed with FACS lysing solution (BD Biosciences). To evaluate cytokine-producing cells, intracellular staining was performed. Lung lymphocytes were resuspended in Iscove’s Modified Dulbecco’s Medium (IMDM; Welgene) supplemented with 10% FBS and stimulated with 10 μM MERS S291 peptide (KYYSIIPHSI) or 10 μg/ml MERS S1 protein (aa 1–725; Sino Biological Inc.) in the presence of Brefeldin A (eBioscience, San Diego, CA) and recombinant human IL-2 (BioLegend) for 5 h at 37°C. After stimulation, these cells were initially stained with anti-CD8a [53–6.7], anti-CD4 [RM4-5], and anti-CD44 [IM7] (BioLegend) for 30 min at 4°C and then fixed. The fixed cells were permeabilized with BD Perm/Wash buffer (BD Biosciences) for 15 min at RT and stained with anti-IFN-γ [XMG1.2] (BioLegend) for 40 min at RT. To distinguish between lung-localized and circulating T cells, immunized mice were intravenously injected with 2 μg of APC-conjugated anti-CD45 antibody (clone 30-F11; BioLegend) in 200 μl PBS and sacrificed after 5 min. Lymphocytes were isolated from lung and spleen as described previously [41]. Subsequently, cells were stained with anti-CD8 [53–6.7], anti-CD69 [H1.2F3], anti-CD103 [2E7] (BioLegend), and K^d^/S291 tetramer. To identify dead cells, the cells were not fixed and subsequently stained with DAPI (BioLegend) for 15 min at RT before analysis on a flow cytometer. Data on all the stained cells were acquired using BD LSR Fortessa (BD Biosciences), which were analyzed using FlowJo software (TreeStar Inc., Ashland, OR).

### Statistical analysis

All data are presented as mean ± standard error of the mean (SEM; n = 4). Statistical differences between two means were analyzed using Two-tailed Student’s *t* test. For comparisons of multiple groups, one-way analysis of variance (ANOVA) or two-way ANOVA along with a Bonferroni post-test were used as appropriate. Statistical significance was considered when *P* values were <0.05.

## Results

### Generation of recombinant adenovirus-based vaccines expressing the Spike gene of MERS-CoV

**Fig 1:**
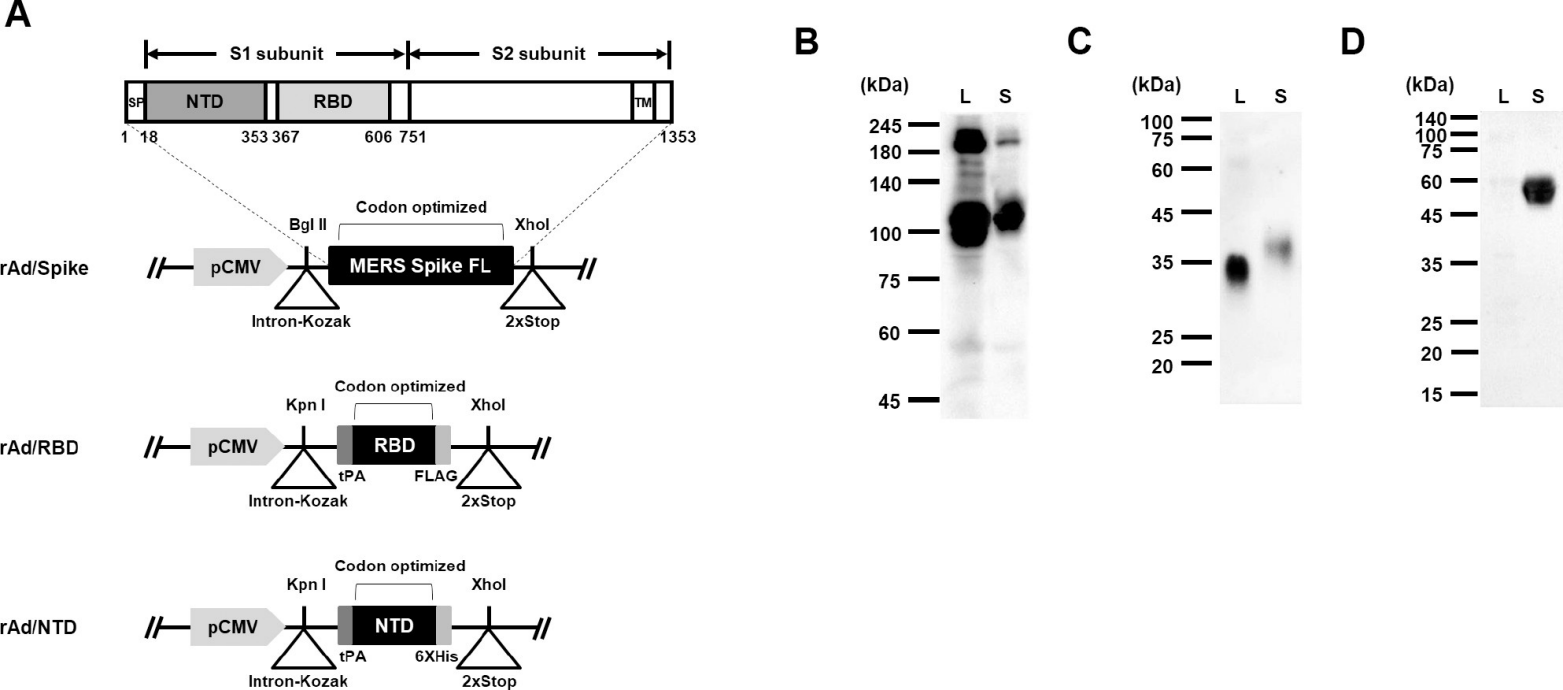
Construction and characterization of rAd-based vaccines. (A) Schematic representation of rAd/Spik, rAd/RBD, and rAd/NTD. A shuttle vector carrying the codon-optimized full-length MERS-CoV Spike gene, RBD gene, and NTD gene was constructed, as shown in the diagram. The vectors were used to generate recombinant replication-deficient adenovirus rAd/Spike, rAd/RBD, and rAd/NTD by homologous recombination with adenoviral genomic DNA. Numbers indicate amino acids. SP, signal peptide; NTD, N-terminal domain; RBD, receptor-binding domain; TM, transmembrane domain. (B-D) Spike, RBD, and NTD expressions in the lysate (L) and supernatant (S) of HEK293 cells infected with (B) rAd/Spike, (C) rAd/RBD, and (D) rAd/NTD, respectively. The expressions of Spike and RBD were confirmed via immunoblotting assay using MERS-CoV spike protein S1 (aa 1–725) rabbit polyclonal antibody. NTD expression were confirmed by immunoblotting assay with anti-rAd/Spike immune serum.

We designed and generated three recombinant adenoviruses as vaccine candidates containing codon-optimized full-length Spike, RBD, or NTD region of the Spike. The coding sequences of full-length Spike (1–1353), RBD (367–606), and NTD (18–353) were cloned into the shuttle vector under the control of the cytomegalovirus (CMV) promoter and subsequently inserted into the adenovirus genome via homologous recombination. The construction schemes of the recombinant replication-defective adenoviruses (designated rAd/Spike, rAd/RBD, and rAd/NTD) were shown in Fig 1A. To confirm transgene expression, HEK293 cells were infected with rAd/Spike, rAd/RBD, or rAd/NTD and Western blotting was performed. Western blot performed using MERS-CoV S1-specific antibody revealed two prominent bands at ~200 kDa and ~120 kDa for the culture supernatant and lysate of rAd/Spike-infected cells (Fig 1B). We considered the protein species with a higher molecular weight of 200 kDa to be the glycosylated S1/S2 Spike protein and the species with a lower molecular weight of ~120 kDa to be the S1 subunit of the Spike protein cleaved by host cell protease [19,33,36,42,43]. The RBD domain was detected as a single band with a molecular weight of 35–40 kDa in the lysate and culture supernatant of rAd/RBD-infected cells (Fig 1C). The molecular weight of RBD increased slightly in the culture supernatant, which might be due to the different patterns of glycosylation between the culture supernatant and the lysate [44]. The single band of ~50 kDa was observed only in the culture supernatant of rAd/NTD-infected HEK293 cells by in-house rAd/Spike-immunized mouse serum (Fig 1D).

### Humoral and cellular responses induced by immunization with candidate MERS-CoV vaccines

**Fig 2:**
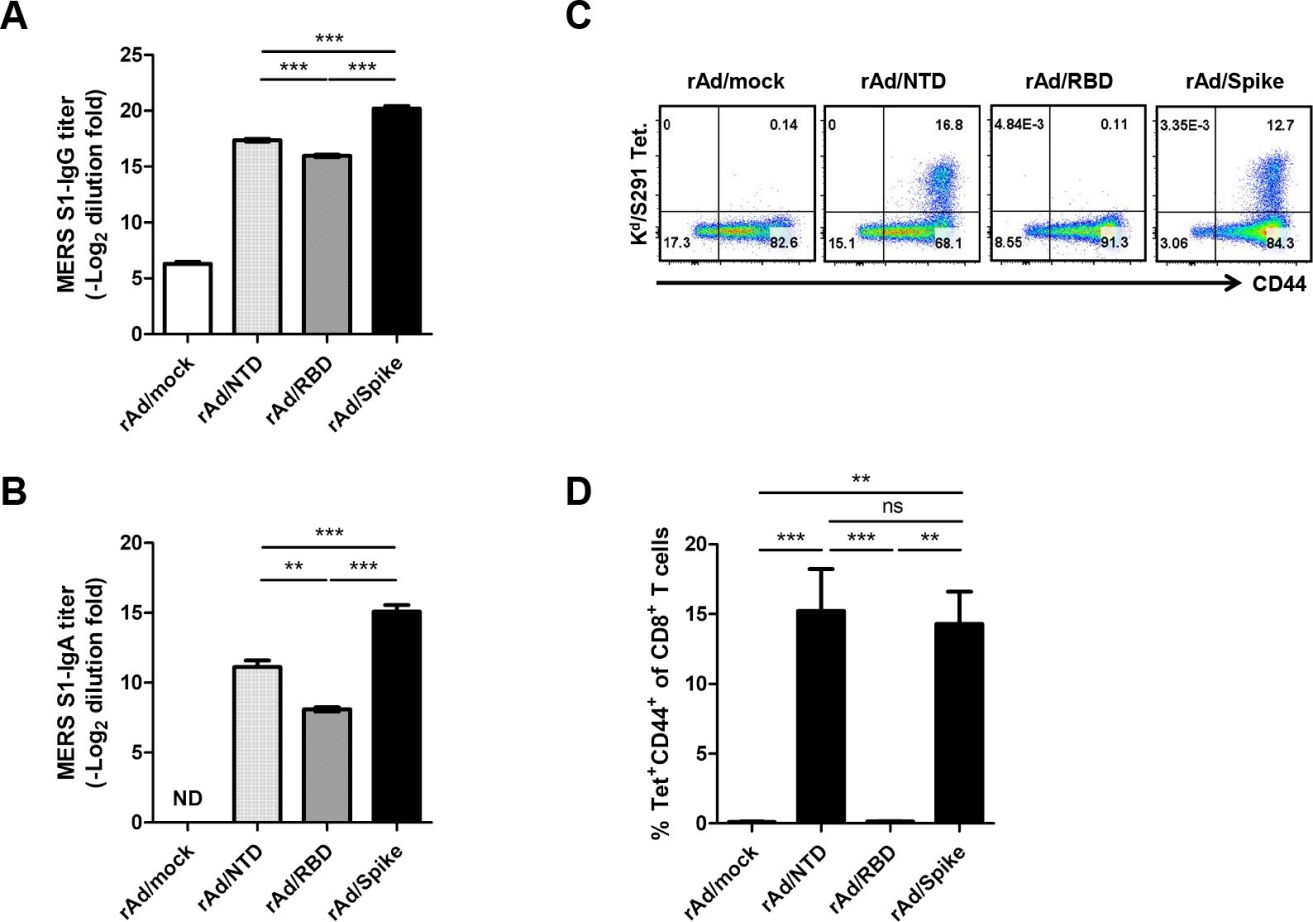
Immunization with rAd/NTD, rAd/RBD, or rAd/Spike elicits humoral and CD8 T cell immune responses. Female BALB/c mice (4 mice/group) were immunized twice at week 0 and 2 with 1×10^7^ PFUs of rAd/NTD, rAd/RBD, or rAd/Spike via the intranasal (IN) route, whereas control animals received 1×10^7^ PFUs of rAd/mock. Sera and BALFs were collected for measuring antibody responses 7 days after the second immunization. (A) Systemic MERS S1-specific serum IgG antibody titers were determined by ELISA. (B) Mucosal MERS S1-specific IgA antibody titers in BALFs were measured by ELISA. (C) On day 7 after the last immunization, lung cells were harvested from mice and stained with K^d^/S291 tetramer, anti-CD8, and anti-CD44 antibody. The frequency of K^d^/S291 tetramer-positive and CD44-positive cells among the total lung CD8-positive cell population is indicated in the upper right quadrant. (D) The average percentages of K^d^/S291 tetramer-specific CD8 T cells in the lungs. Data are representative of at least two independent experiments with similar results and average SEM value of four mice. ns, not significant; ***p<0.001; **p<0.01.

To compare the immunogenicity of our recombinant adenoviral vaccine candidates, BALB/c mice were immunized twice on day 0 and day 14 with rAd/NTD, rAd/RBD, rAd/Spike, or control adenovirus (rAd/mock) via IN route. Mice were sacrificed at 7 days after boost immunization to analyze both humoral and cellular immune responses. All candidate vaccines induced significant S1-specific systemic IgG responses in sera as opposed to the negative control rAd/mock vaccine (Fig 2A). Notably, the rAd/Spike-immunized group induced the highest S1-specific serum IgG responses among the three groups.

Mucosal IgA in the respiratory tract plays critical roles in host defense against respiratory virus infections [45]. Thus, we measured the Spike-specific mucosal IgA antibody responses in BALFs. S1-specific IgA was detected in all vaccination groups but not in the control group (Fig 2B). Among the three vaccination groups, the rAd/Spike group elicited the highest levels of S1-specific IgA response, and the rAd/NTD group showed a higher titer than that shown by the rAd/RBD group.

Next, to examine the ability of our candidate vaccines to induce cellular immune responses, mice were sacrificed at 7 days after boost injection, which is the peak of CD8 T-cell responses,and their lung cells were harvested. Lung lymphocytes were stained with K^d^/S291 tetramer to quantify Spike-specific CD8 T cells. Previous studies have reported that CD8 T-cell responses to the MERS-CoV Spike protein were dominantly directed toward S291 (KYYSIIPHSI) epitope [46]. Other studies also screened epitopes in the RBD region to measure antigen-specific CD8 T-cell responses, but the frequency of IFN-γ-producing CD8 T cells responding to other peptides seems to be either undetectable or very low [46,47]. Therefore, we employed the S291 immunodominant epitope to evaluate the Spike-specific CD8 T-cell responses. Significant percentages of K^d^/S291 tetramer-positive CD8 T cells were detected in the lungs of rAd/NTD- or rAd/Spike-immune mice but not in the lungs of the mice in the rAd/mock or rAd/RBD group (Fig 2C and 2D). S291-specific CD8 T cells were not observed in rAd/RBD-immunized mice, because RBD (aa residues from 367 to 606) does not contain the S291 epitope. To assess the levels of effector CD8 and CD4 T cells secreting IFN-γ, lung lymphocytes were stimulated with S291 peptide or MERS S1 protein, respectively. IFN-γ-producing CD8 T cells were also increased significantly in the rAd/Spike and rAd/NTD groups, and IFN-γ-producing CD4 T cells were increased significantly in the rAd/Spike group (S1 Fig).

**Fig 3:**
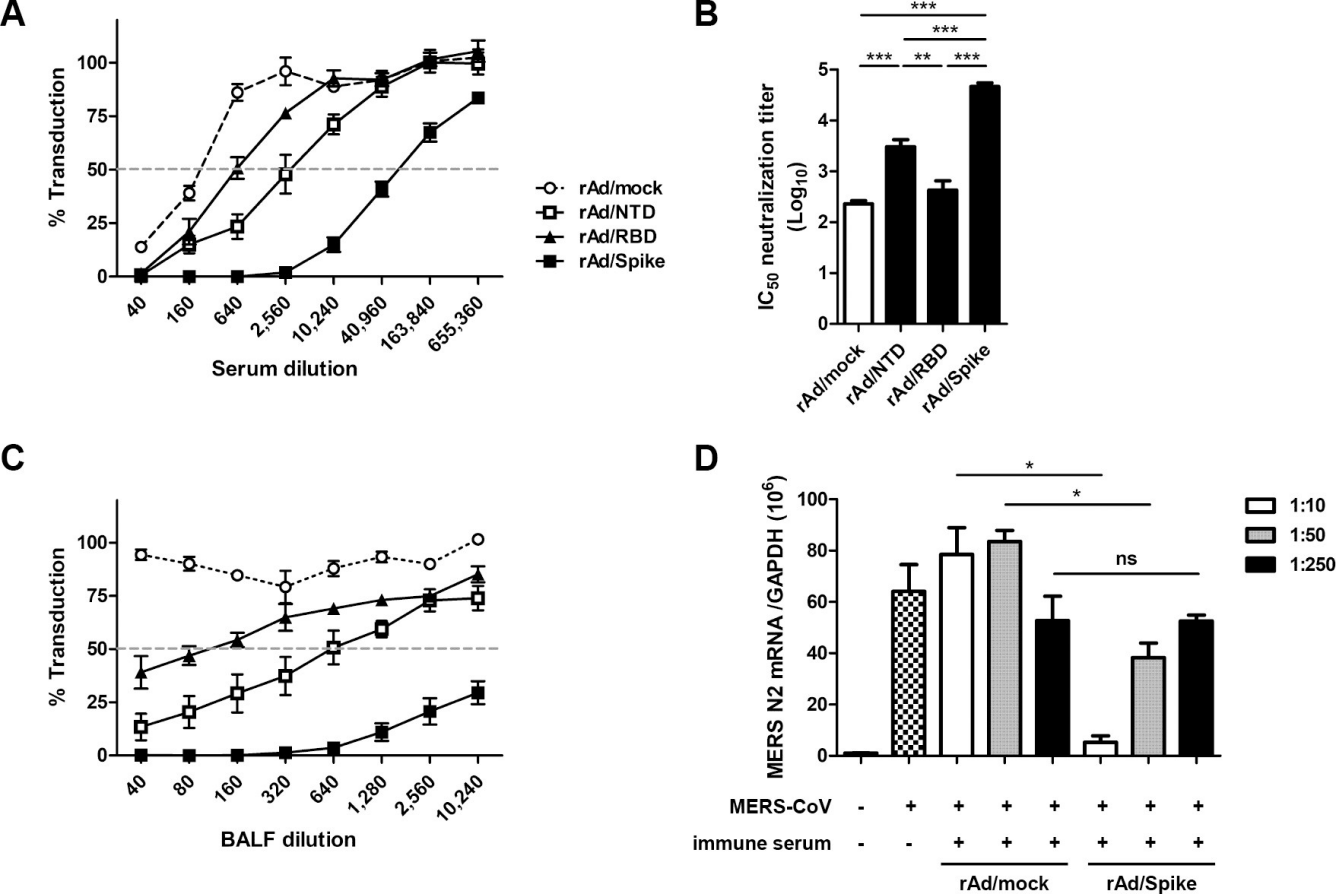
The neutralizing capacity directed against MERS Spike pseudotyped virus. Groups of mice were immunized as indicated in Fig 2. On day 7 after the last immunization, sera and BALFs were collected from each group. Serially diluted sera and BALFs were evaluated for their neutralizing activity against MERS Spike pseudotyped virus in Huh 7.5 cells. The transduction percentage was calculated by measuring GFP expression in comparison with the expression in the control pseudovirus-infected cells. The dotted horizontal line indicates 50% neutralization. (A) Neutralization activities in serially diluted sera. (B) The IC_50_ (50% inhibitory concentration) neutralization titers in the sera. (C) The neutralization activities in serially diluted BALFs. Data are representative of at least two independent experiments with similar results and average SEM value of four mice. (D) NHNE cells (10^6^ cells/well) were inoculated with 300 μl of virus solution (MERS-CoV 10μl/PBS 10 ml) and *N2* gene level of MERS-CoV was assessed at 1 day post-infection using real-time PCR. To prove the effect of neutralizing antibody for MERS-CoV spike protein, targeted antibody of MERS-CoV spike protein was diluted to 1:10, 1:50, 1:250 and the diluted antibodies were mixed with the virus solution at a ratio of 1:1 and maintained for 1 hour before infection to NHNE cells. Then, *N2* gene level of MERS-CoV was assessed at 1 day post-infection using real-time PCR. The same experiments were carried out using mock antibody and the result was compared with that of targeted antibody for MERS-CoV spike protein. ns, not significant; ***p<0.001; **p<0.01; *p<0.05.

Subsequently, neutralizing activities of the immune sera and BALFs were evaluated using MERS-CoV Spike pseudotyped particles. Among the candidate vaccine groups, the rAd/Spike-immune mice exhibited the highest levels of neutralizing antibody in both sera and BALFs, followed by mice in the rAd/NTD group (Fig 3A–3C). However, rAd/RBD group exhibited no significant difference in neutralizing activity compared with the rAd/mock control group. To further confirm the neutralizing activity of the rAd/Spike-induced antibody in an authentic MERS-CoV infection of human primary respiratory cells, MERS-CoV were preincubated with the immune sera from rAd/Spike and rAd/mock group before infection of NHNE cells. Subsequently the viral replication levels were measured by quantitative RT-PCR. As shown in Fig 3D, pre-treatment of rAd/Spike-immune sera significantly inhibited MERS-CoV replication while rAd/Mock sera did not. Taken together, these results demonstrate that immunization with rAd/Spike is the most efficient for eliciting both neutralizing antibody responses in serum and BALF and T-cell responses.

### Immunization with rAd/Spike induces neutralizing antibodies targeting the spike glycoprotein within and outside RBD

**Fig 4:**
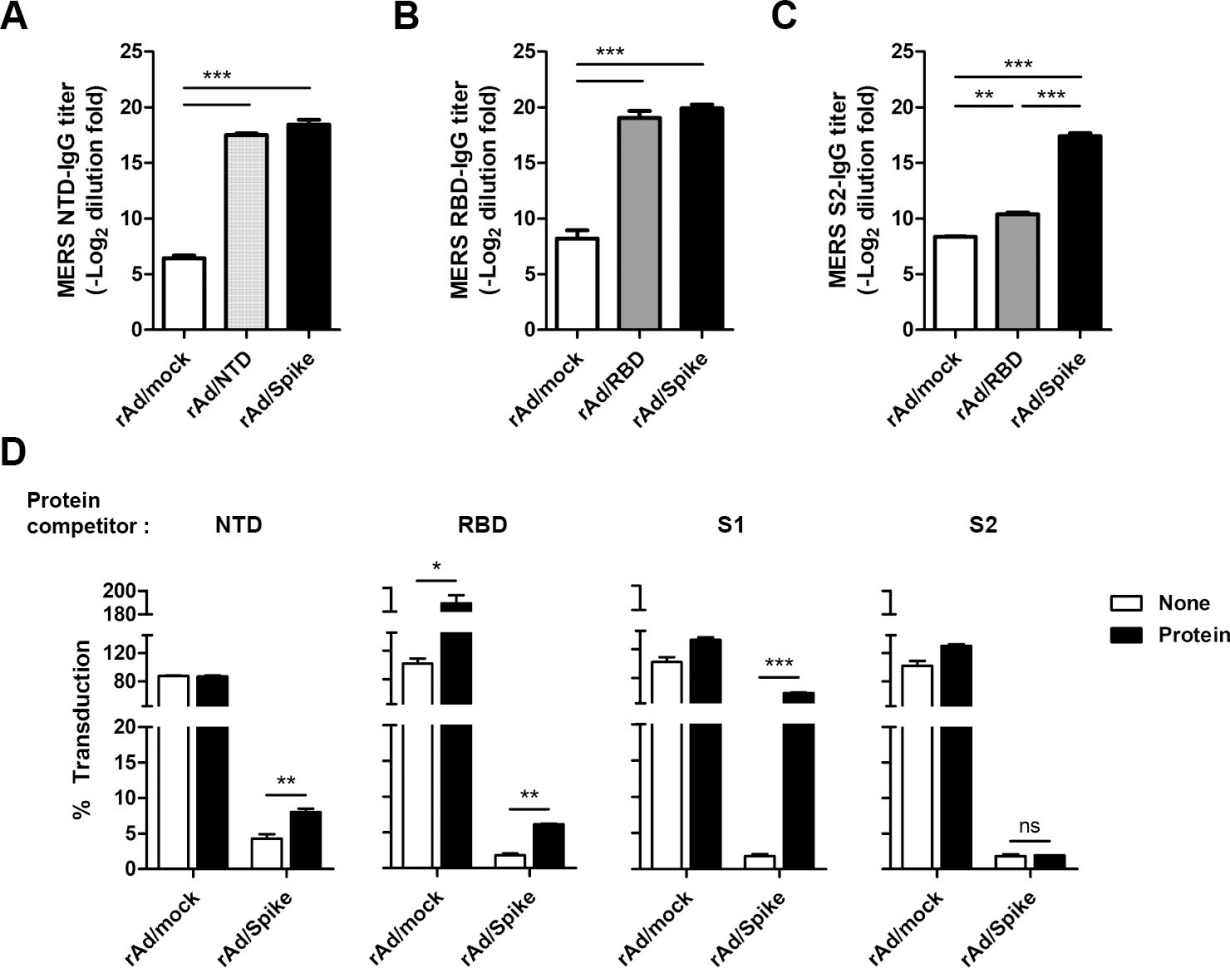
Characterization of neutralizing antibody responses induced by rAd/Spike. Groups of mice were immunized as indicated in Fig 2. On day 7 after the last immunization, sera were collected from immune mice. (A) The NTD-specific, (B) RBD-specific, and (C) S2-specific IgG antibody responses were measured by ELISA. NTD, RBD, and S2 proteins were used as coating antigens (200 ng/well), and goat anti-mouse IgG-HPR was used as the detection antibody. (D) Protein competition neutralization assay. Sera collected from rAd/Spike immune mice were diluted at a ratio of 1:640 and were assayed for neutralization of MERS Spike pseudotyped virus in the presence of soluble NTD, RBD, S1, or S2 proteins at a concentration of 10 μg. Data are represented as mean ± SEM results of four mice from at least two independent experiments. ns, not significant; ***p<0.001; **p<0.01; *p<0.05.

Recent studies have shown that neutralizing antibodies could also be directed against non-RBDs, such as NTD, besides RBD [26,48]. Our results also indicate that NTD can elicit neutralizing antibody responses after immunization. Therefore, we further investigated the regional specificity of neutralizing activities induced by immunization with rAd vaccines. First, serum IgG responses were analyzed by ELISA using NTD, RBD, or S2 protein as coating antigens. The rNTD protein was prepared and purified using a baculovirus expression system (S2 Fig). Both rAd/NTD and rAd/Spike groups exhibited significantly higher levels of NTD-specific serum IgG than the control group, and there was no significant difference in NTD-specific serum IgG levels between the rAd/NTD and rAd/Spike groups (Fig 4A). Both the rAd/RBD and rAd/Spike groups exhibited significantly higher levels of RBD-specific serum IgG than the negative control group (Fig 4B). As expected, only rAd/Spike group showed significantly higher S2-specific serum IgG responses than the control group (Fig 4C).

To investigate the target domains mainly involved in neutralization, we compared changes in the neutralizing activity of sera collected from rAd/Spike group using protein competitors. Sera diluted at 1:640 were pre-incubated with 10 μg of NTD, RBD, S1, or S2 protein at 37°C for 1 h; neutralization assay was subsequently performed as shown in Fig 3. As shown in Fig 4D, neutralization activity of immune sera was significantly reduced by pre-incubation with NTD, RBD, and S1 protein competitors. Especially, competition with the S1 protein inhibited neutralizing activity by ~60%, while NTD and RBD competitors inhibited neutralization activity by ~8% and ~6%, respectively. In contrast, soluble S2 protein competitor did not have any effect on neutralization activity. These results demonstrate that rAd/Spike can induce neutralizing antibody responses targeting various regions of S1 including NTD and RBD. This also suggests that employing rAd/Spike might be better vaccination strategy in eliciting broader spectrum of neutralizing antibody responses.

### Comparison between humoral immune responses induced by rAd/Spike via intranasal, intramuscular, and sublingual routes

**Fig 5:**
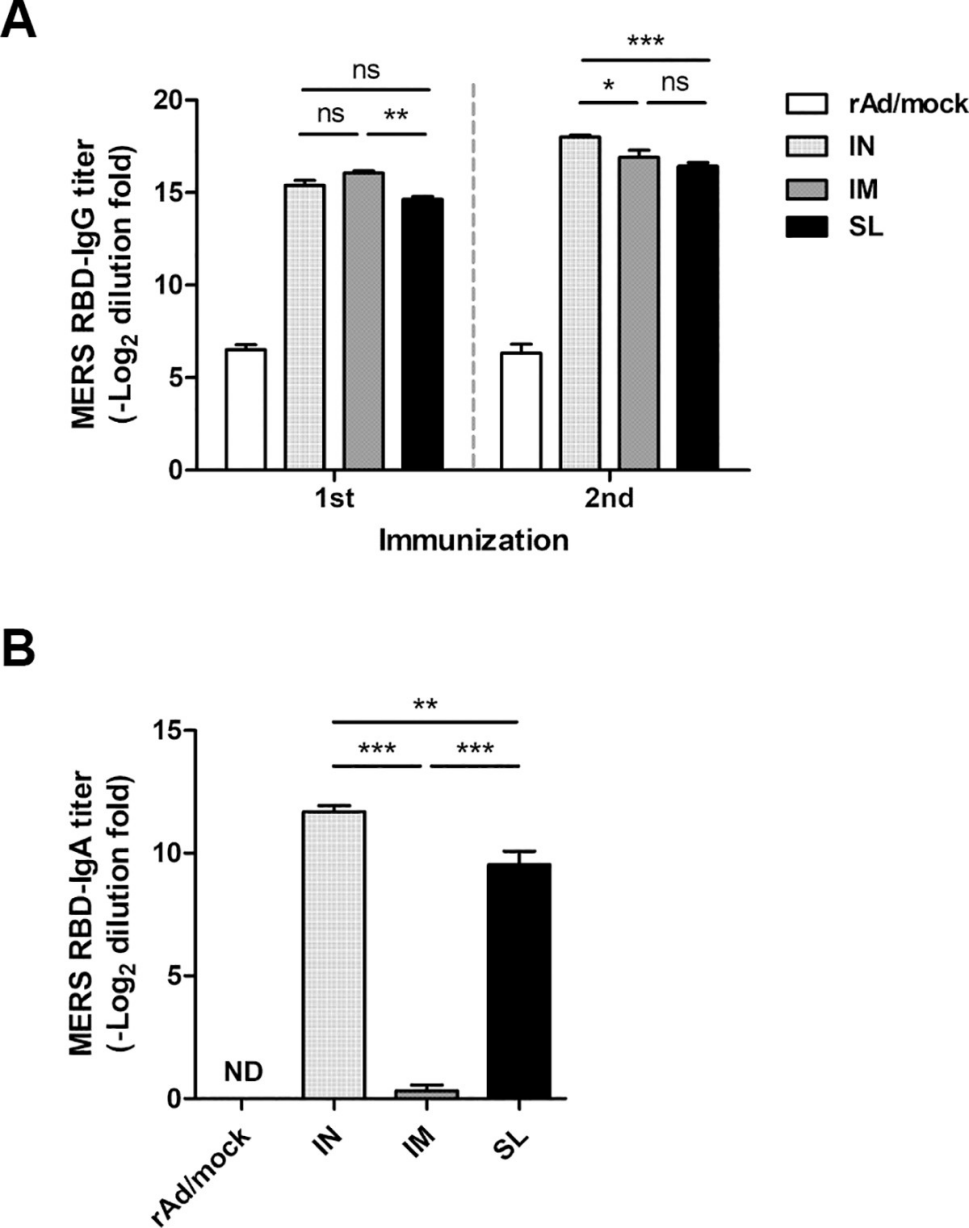
Detection of humoral immunity induced by rAd/spike via different immunization routes. Female BALB/c mice were immunized twice at week 0 and 2 with 1×10^8^ PFU of rAd/Spike via IN, sublingual (SL), or intramuscular (IM) route. Control mice were immunized intranasally with 1×10^8^ PFU of rAd/mock. Sera were collected 2 weeks after each vaccination for measuring antibody responses. BALFs were harvested 2 weeks after the last immunization (A) Systemic RBD-specific IgG titers in sera were measured using ELISA. (B) Mucosal RBD-specific IgA titers in BALFs were measured using ELISA. The data are presented as mean (Log_2_ endpoint titers) ± SEM values of four mice per group. ns, not significant; ***p<0.001; **p<0.01; *p<0.05; ND, not detected.

Previously, we showed that among all vaccine candidates, rAd/Spike possesses the strongest immunogenicity for inducing humoral and cellular immune responses. Then, we investigated the best route for immunization by injecting mice with rAd/Spike via IN, IM, or SL route. Sera were collected 2 weeks after each immunization and measured for specific IgG by ELISA. Significant Spike-specific IgG levels were detected regardless of the immunization route (Fig 5A). Notably, IN immunization induced the highest IgG antibody responses among the groups. After the boost immunization, significant Spike-specific IgA levels were detected in BALFs of mice from IN and SL groups, but not in those from the IM group (Fig 5B), and the response was also highest in the IN group.

**Fig 6:**
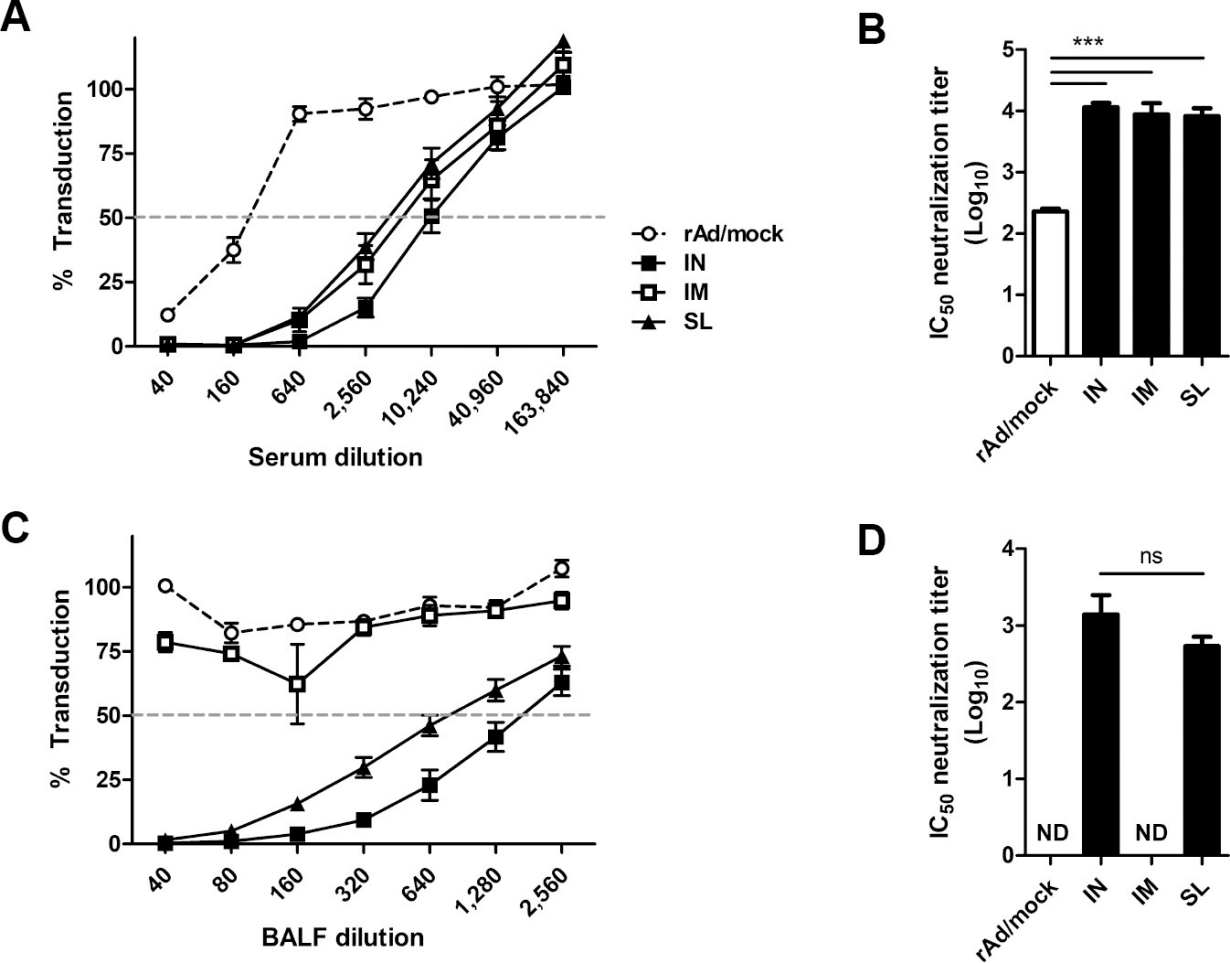
Comparison of neutralizing activity induced by rAd/spike via different immunization routes. Groups of mice were immunized as indicated in Fig 5. Two weeks after last immunization, sera and BALFs were collected from immune mice and heat-inactivated at 56°C for 30 min before detecting neutralizing antibodies. (A) The neutralizing antibody titers in serially diluted sera were measured via MERS-CoV pseudovirus neutralization assay in Huh 7.5 cells. (B) IC_50_ neutralization titer in the serum. (C) Neutralizing antibody titers in serially diluted BALFs. (D) IC_50_ neutralization titer in BALFs. The data are presented as mean ± SEM values of four mice per group. ns, not significant; ***p<0.001.

To investigate whether the immunization route affects neutralizing antibody responses, the neutralizing activity of sera and BALFs collected from each group was analyzed using the pseudotyped virus. All immunization routes generated significant serum-neutralizing antibody levels as opposed to the rAd/mock control group (Fig 6A and 6B). However, no neutralization activity was observed in BALF of mice immunized via IM route; this finding was consistent with mucosal IgA response shown in Fig 6. IC_50_ neutralization titer did not show statistically significant differences between IN and SL immunization. Taken together, these results indicate that mucosal immunization of rAd/Spike vaccine can induce both systemic and mucosal neutralizing antibodies, while IM immunization could only elicit systemic responses.

### Intranasal immunization of rAd/Spike establishes CD8 T_RM_ cells in the airway and lung parenchyma

**Fig 7:**
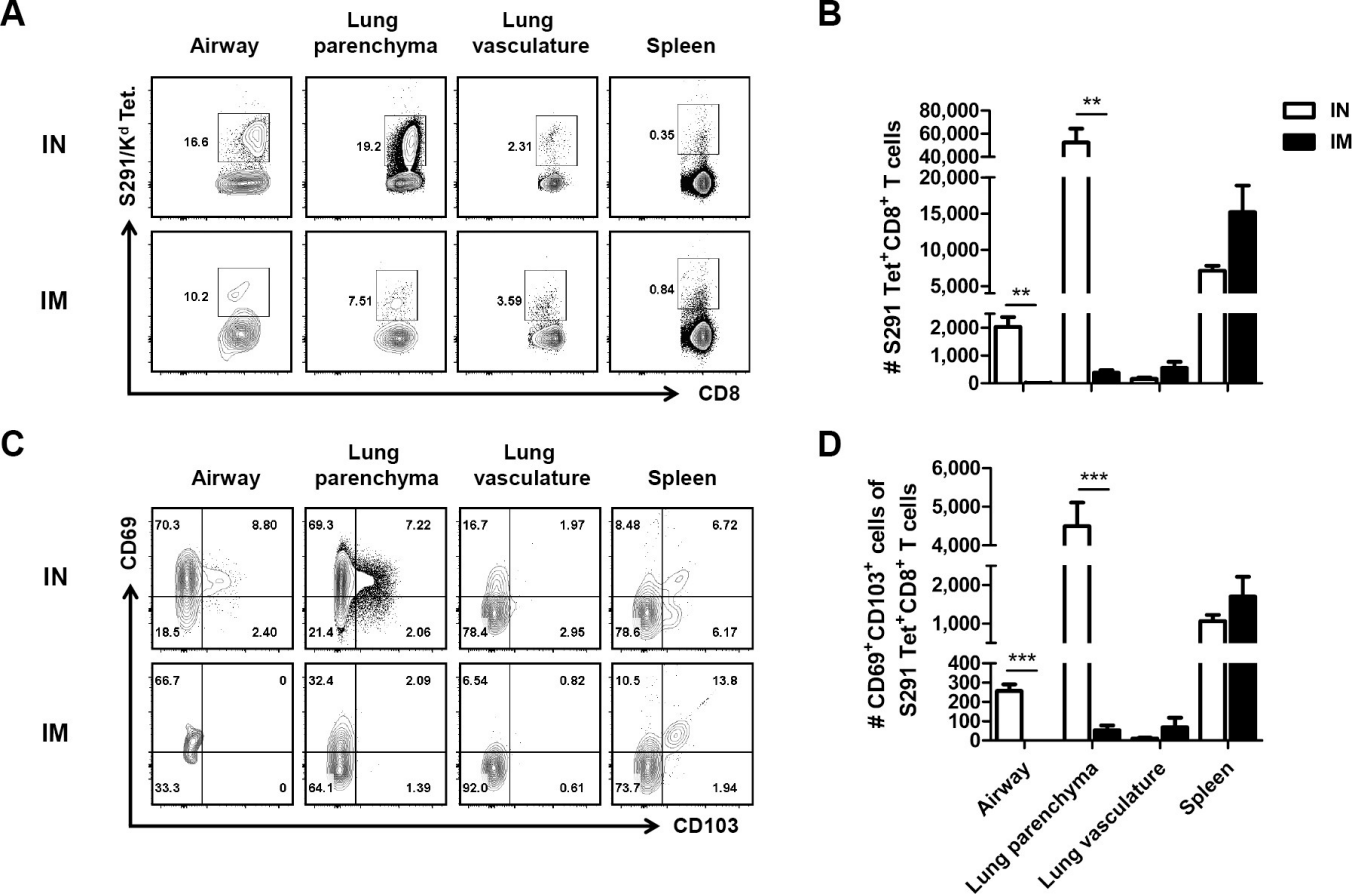
Generation of CD8 T_RM_ cells in the airway and lung parenchyma following intranasal immunization with rAd/Spike but not following intramuscular immunization. On day 30 after immunization with 1×10^8^ PFUs of rAd/Spike via the IN or IM route, mice were injected intravenously with 2 μg of anti-CD45 antibody and sacrificed after 5 min. (A) Representative staining of K^d^/S291 tetramer-specific CD8 T cells distributed in the airway, lung parenchyma, lung vasculature, and spleen of mice immunized via the IN or IM route. (B) The numbers of K^d^/S291 tetramer-positive CD8 T cells in the airway, lung parenchyma, lung vasculature, and spleen 30 days after IN (white bar) or IM (black bar) immunization. (C) Representative staining of CD69 and CD103 expressed on K^d^/S291 tetramer-positive CD8 T cells, which are distributed in the airway, lung parenchyma, lung vasculature, and spleen of mice on day 30 after IN or IM immunization. (D) The number of K^d^/S291 tetramer-specific CD8 T cells expressing CD69 and CD103 in the airway, lung parenchyma, lung vasculature, and spleen 30 days after IN or IM immunization. Data are presented as mean ± SEM values of four mice per group. ***p<0.001; **p<0.01.

Lung-resident memory T (T_RM_) cells reportedly mediate protection against respiratory virus infections, and mucosal immunization is thought to generate T_RM_ cells in the lung [49–51]. To investigate the influence of immunization route in the establishment of lung T_RM_ cells, BALB/c mice were administered IN or IM vaccine containing rAd/Spike at a concentration of 1×10^8^ PFUs. At a memory time point (day 30), mice were intravenously injected with anti-CD45-APC antibody and sacrificed after 5 min to discriminate circulating cells from lung-localized cells [52]. As shown in S3 Fig, cells stained with anti-CD45 Ab were identified as circulating T cells in blood, and cells protected from *in vivo* labeling were confined to the lung parenchyma. At this time point, mice immunized via IN route had significantly higher frequencies and numbers of K^d^/S291 tetramer-positive CD8 T cells in the airway and lung parenchyma compared with IM-immunized mice (Fig 7A and 7B). In contrast, there were no significant differences in the numbers of K^d^/S291 tetramer-positive CD8 T cells in the lung vasculature and spleen between IN and IM immunization groups.

We subsequently assessed the co-expression of CD103 and CD69 as the canonical makers of lung T_RM_ [53]. A majority of K^d^/S291 tetramer-positive CD8 T cells in the airway and lung parenchyma in IN-immunized mice were CD69^+^ or CD69^+^CD103^+^, and their frequencies were significantly higher than IM-immunized mice (Fig 7C and 7D). Taken together, these results show that IN immunization is more efficient in establishing antigen-specific CD8 T_RM_ cells in the airway and lung parenchyma than IM immunization.

### Immunization with rAd/Spike elicits long-lasting neutralizing antibody responses

**Fig 8:**
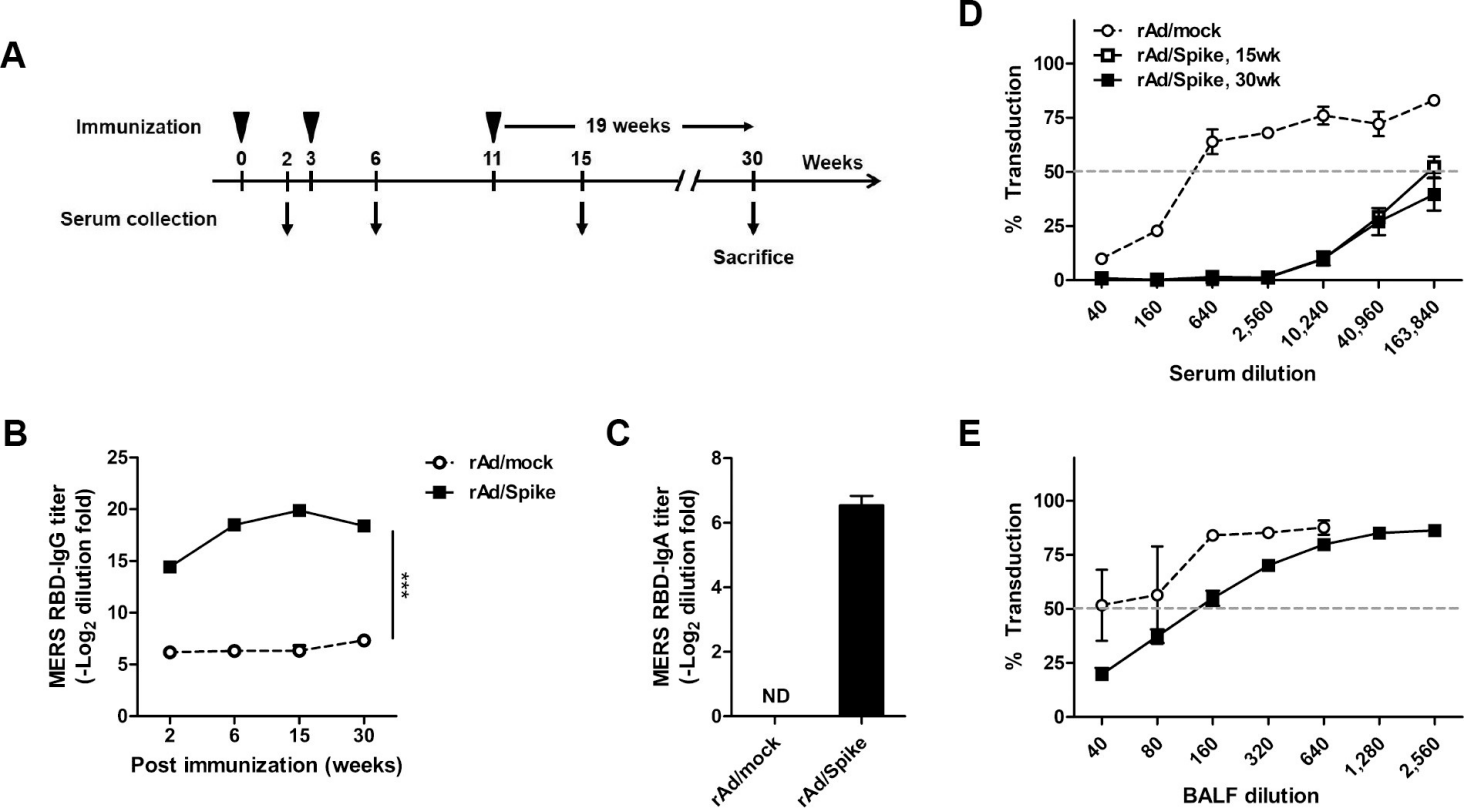
Detection of long-lasting humoral immune responses elicited rAd/Spike immunization. Female Balb/c mice were immunized three times at 0, 3, and 11 weeks with 1×10^7^ PFU of rAd/Spike via IN route. Sera were collected for measuring antibody responses at 2, 6, 15, and 30 weeks. Control mice were immunized intranasally with 1×10^8^ PFU of rAd/mock. (A) Immunization scheme. (B) Systemic RBD-specific IgG antibody titers were measured by ELISA after each immunization. (C) Mucosal RBD-specific IgA antibody titers in BALFs were measured 19 weeks after the last immunization by ELISA. (D) Neutralization antibody responses in sera were measured via MERS-CoV pseudovirus neutralization assay at 15 and 30 weeks. (E) Neutralizing activities in BALFs at 19 weeks after the third immunization. Data are presented as mean ± SEM values of four mice per group. ***p<0.001.

The persistence and maintenance of immune responses following vaccination is important for vaccine efficacy [54]. Thus, we assessed whether rAd/Spike immunization could induce long-term antibody responses. BALB/c mice were inoculated three times (weeks 0, 3, and 11) with 1×10^7^ PFUs of rAd/Spike via IN route, and sera were collected at 2, 6, 15, and 30 weeks; the specific antibody responses were subsequently measured by ELISA (Fig 8A). Spike-specific serum IgG and mucosal IgA titers gradually increased with each immunization and maintained until 30 weeks with no obvious decline (Fig 8B and 8C).

To evaluate neutralizing activities, the assay was performed using sera collected at 15 and 30 weeks as well as BALFs obtained at 30 weeks. In agreement with IgG responses, serum neutralizing activities at 30 weeks were similar to those at 15 weeks (Fig 8D). The absolute titer of neutralization in BALF at 30 weeks was lower than that at an earlier time point shown in Fig 3, but remained at a significant level in comparison with the control (Fig 8E). Overall, the results demonstrate that IN immunization with rAd/Spike could induce long-lasting neutralizing antibodies, which might be beneficial for protection against MERS-CoV infection.

## Discussion

Since the emergence and spread of MERS, the development of safe and effective vaccines against MERS-CoV is urgently required [7,55]. MERS-CoV Spike protein is the main mediator of virus entry into the target cells [17,23]. Therefore, Spike protein is one of key targets for the development of vaccines and therapeutics [23]. Several vaccine candidates targeting RBD of the Spike protein have been investigated [22,25,28,29,32,56]. However, it seems that the efficacy of the RBD-based vaccine candidates are limited by the confined breadth of antibody responses and subsequent emergence of escape mutants [27,38,57]. Indeed, it has been shown that several mutations in the RBD region can result in the generation of neutralization escape variants [38,58–60]. Several studies have reported that other regions of Spike protein rather than RBD can also be targets of neutralizing antibodies [26,27,48,61].

In this study, we constructed adenovirus-based vaccine candidates that express NTD, RBD, and full-length Spike and compared immunogenicity and neutralizing activity induced by these candidates. Our results clearly showed that the rAd/Spike vaccine expressing full-length Spike protein is the best candidate in terms of immunogenicity and neutralizing efficacy. It is likely that neutralizing epitopes of the Spike protein were located on non-RBD of the S1 subunit as well as RBD, based on the results of protein competitor experiments (Fig 4). The use of full-length Spike as an immunogen in rAd/Spike vaccine might increase the breadth of the antibody responses which will help to reduce the risk of escape mutations [27,57,62]. However, it is not yet clear whether S2 region also contributes for inducing neutralizing antibodies by rAd/Spike vaccination. S2 subunit protein as a competitor did not reduce the neutralizing activities of the immune sera in the protein competitor experiments (Fig 4), and this might be due to the inappropriate conformation of S2 subunit [27,63]. Further studies are needed to determine whether the S2 subunit also contributes for eliciting neutralizing antibodies.

It is noteworthy that the rAd/Spike vaccine induced systemic IgG, mucosal IgA, and T-cell responses together upon IN immunization. In addition to neutralizing antibodies, virus-specific CD8 T cells also play an important role in MERS-CoV clearance [46]. Indeed, several studies suggest that both virus-neutralizing antibodies and cytotoxic CD8 T cell responses are necessary for complete protection against MERS-CoV infection [29,33,35]. Therefore, the rAd/Spike vaccine expressing the full-length Spike protein rather than the RBD or NTD alone might be most potent one among our vaccine candidates.

Our results also demonstrate that IN immunization induces superior responses compared to IM and SL immunization, in terms of neutralizing efficacy. MERS-CoV infection occurs through respiratory tracts in humans [3,64]. Accordingly, vaccination routes capable of inducing local mucosal immunity such as secretory IgA would be more potent in preventing MERS-CoV infection. In addition, IN immunization with rAd/Spike vaccine induced significantly higher numbers of specific T_RM_ cells in the airway and lung parenchyma compared with IM-immunized mice (Fig 6). It is likely that lung-resident T_RM_ cells mediate effective protective immunity against respiratory infections [53,65]. Thus, IN immunization of rAd/Spike eliciting systemic IgG, secretory IgA, and T_RM_ responses together might be the most promising regimen compared to other immunization routes, and provide superior protection.

In summary, we generated several recombinant adenovirus-based vaccine candidates against MERS-CoV and compared systemic and local humoral responses and cellular responses via different immunization routes. We found that single IN immunization of rAd/Spike successfully induces systemic IgG, secretory IgA, and lung T_RM_ responses and provides long-lasting neutralizing immunity. Therefore, our recombinant adenovirus-based vaccine could be further developed as a safe and efficacious vaccine for preventing MERS-CoV infection and spreading.

## Supporting information

S1 Fig Cytotoxic CD8 T cell responses induced by rAd/NTD, rAd/RBD, and rAd/Spike. BALB/c mice (n = 4 per group) were vaccinated twice at week 0 and 2 with 1×10^7^ PFUs of rAd/NTD, rAd/RBD, or rAd/Spike via the IN route, whereas control animals received 1×10^7^ PFUs of rAd/mock. On day 7 after the last vaccination, mice were sacrificed and lungs were harvested. (A-B) Lung cells were unstimulated or stimulated with S291 peptide in the presence of Brefeldin A for 5 h and then stained with anti-CD8, anti-CD44, and anti-IFN-γ antibody. (A) The average percentages and (B) total number of IFN-γ-expressing CD8 T cells in the lungs. (C-D) Lung cells were also stimulated with S1 protein in the presence of Brefeldin A for 5 h and then stained with anti-CD4, anti-CD44, and anti-IFN-γ antibody. (C) The average percentages and (D) total number of IFN-γ-expressing CD4 T cells in the lungs. Data are representative of at least two independent experiments with similar results and average SEM value of four mice. ***p<0.001; **p<0.01; *p<0.05.
(TIF)

S2 Fig Schematic of MERS-CoV NTD protein and expression of recombinant NTD protein. (A) Schematic representation of recombinant NTD (rNTD) protein. (B) SDS-PAGE of purified rNTD protein expressed via the baculovirus expression system. (C) Western-blot analysis of rNTD protein using anti-rAd/Spike immune antibody.
(TIF)

S3 Fig Representative gating strategy for analyzing resident-memory CD8 T cell populations in the lung. BALB/c mice (n = 4 per group) were immunized with 1×10^8^ PFUs of rAd/Spike via the IN or IM route. On day 30, mice were injected intravascularly with APC-conjugated anti-CD45 antibody and sacrificed after 5 min. BAL as well as the lungs and spleen were harvested and cells were stained with K^d^/S291 tetramer, DAPI, anti-CD8, anti-CD103, and anti-CD69 antibodies. Cells were gated for singlets (FSC-A/FSC-H), lymphocytes (FSC-A/SSC-A), and live cells (FSC-A/DAPI^−^). Then, lung cells were discriminated as follows: lung parenchyma cells (K^d^/S291 Tet^+^CD8^+^CD45^–^) and lung vasculature cells (K^d^/S291 Tet^+^CD8^+^CD45^+^).
(TIF)
