# Supplementary figures and images for: Superior immune responses induced by intranasal immunization with recombinant adenovirus-based vaccine expressing full-length Spike protein of Middle East respiratory syndrome coronavirus
Source: PLoS One. 2019 Jul 22;14(7):e0220196. doi: 10.1371/journal.pone.0220196 (PMC6645677; doi:10.1371/journal.pone.0220196)

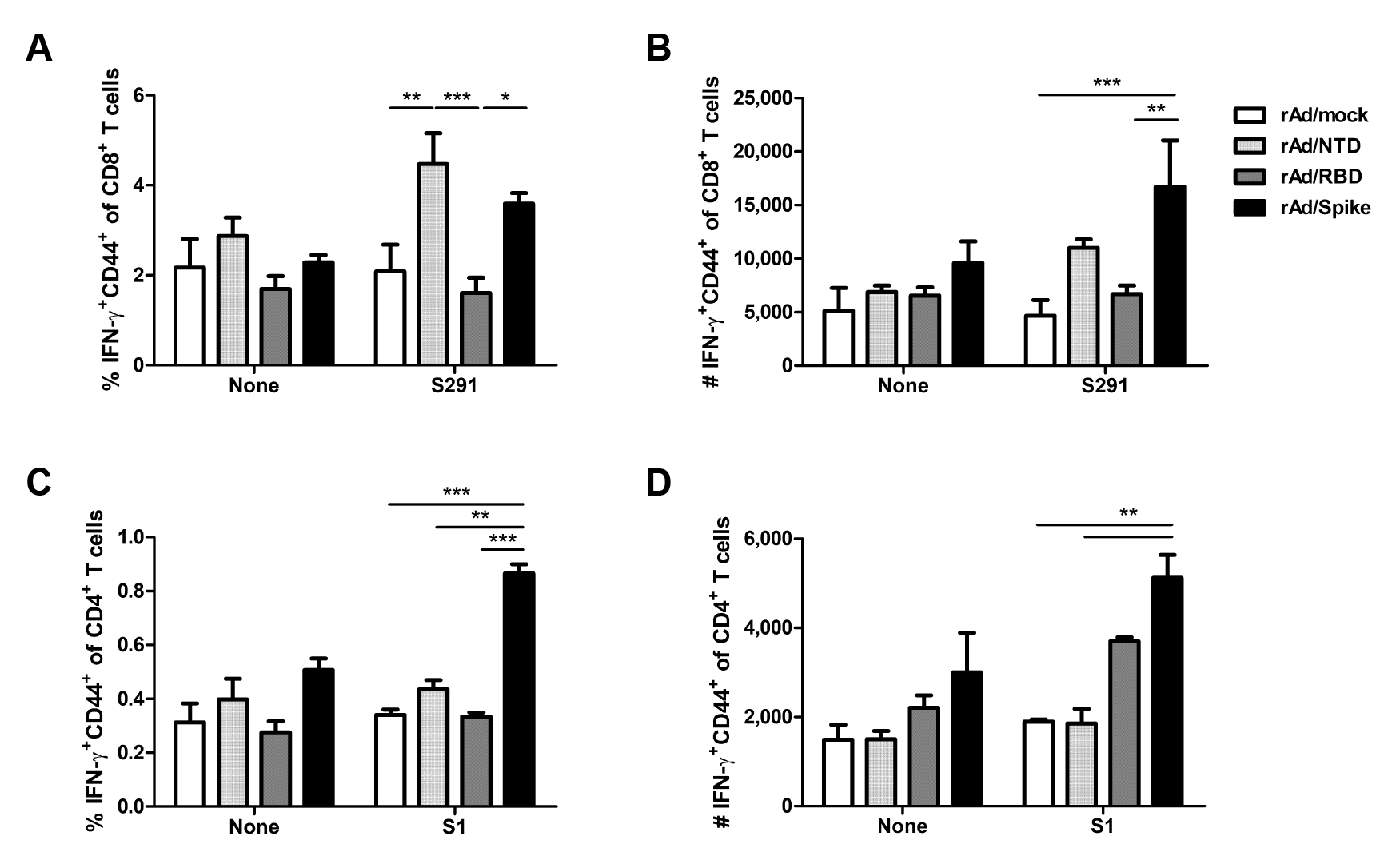

Supplement: S1 Fig — BALB/c mice (n = 4 per group) were vaccinated twice at week 0 and 2 with 1×10^7^ PFUs of rAd/NTD, rAd/RBD, or rAd/Spike via the IN route, whereas control animals received 1×10^7^ PFUs of rAd/mock. On day 7 after the last vaccination, mice were sacrificed and lungs were harvested. (A-B) Lung cells were unstimulated or stimulated with S291 peptide in the presence of Brefeldin A for 5 h and then stained with anti-CD8, anti-CD44, and anti-IFN-γ antibody. (A) The average percentages and (B) total number of IFN-γ-expressing CD8 T cells in the lungs. (C-D) Lung cells were also stimulated with S1 protein in the presence of Brefeldin A for 5 h and then stained with anti-CD4, anti-CD44, and anti-IFN-γ antibody. (C) The average percentages and (D) total number of IFN-γ-expressing CD4 T cells in the lungs. Data are representative of at least two independent experiments with similar results and average SEM value of four mice. ***p<0.001; **p<0.01; *p<0.05. (TIF) [file pone.0220196.s001.tif]

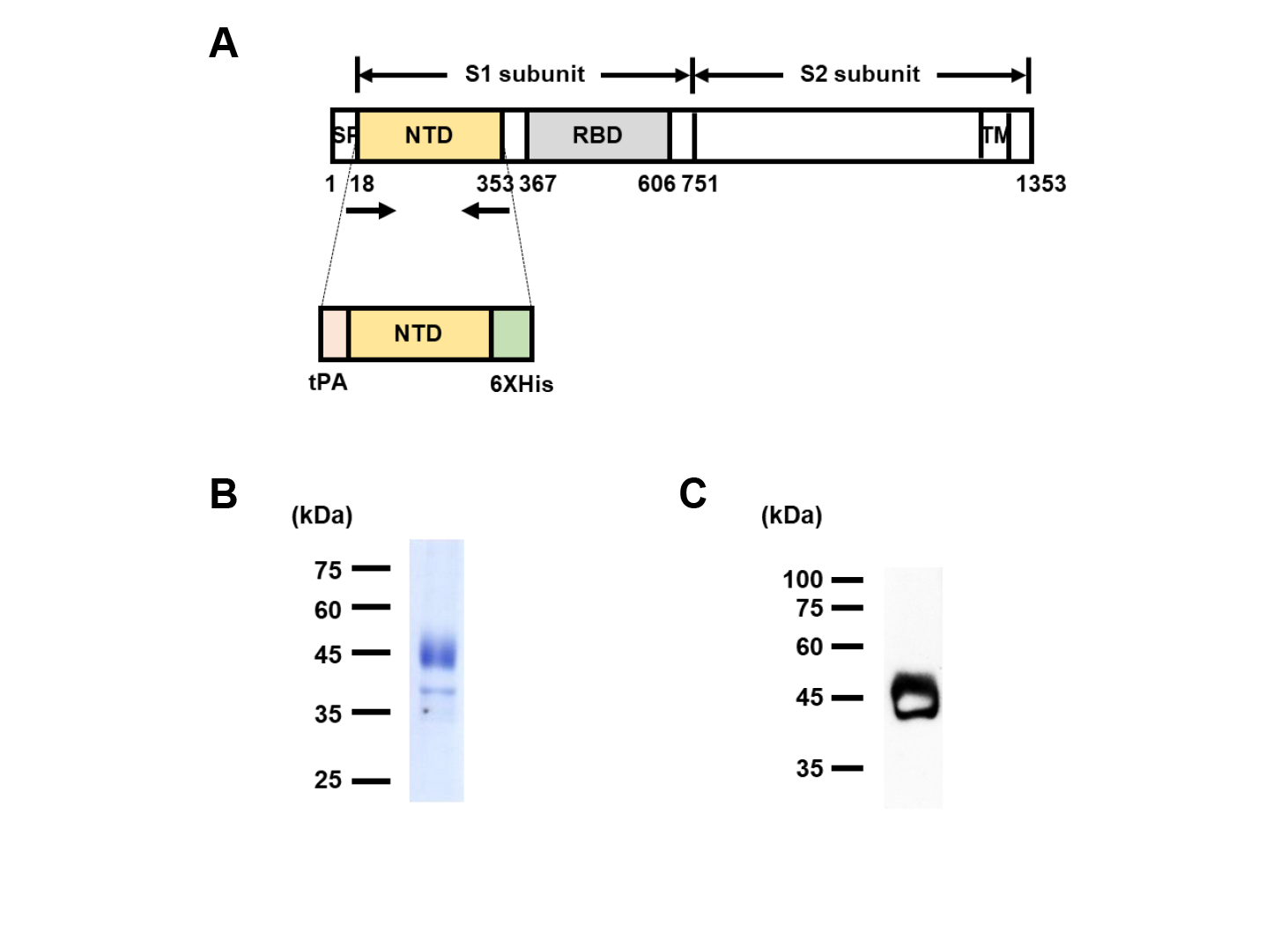

Supplement: S2 Fig — (A) Schematic representation of recombinant NTD (rNTD) protein. (B) SDS-PAGE of purified rNTD protein expressed via the baculovirus expression system. (C) Western-blot analysis of rNTD protein using anti-rAd/Spike immune antibody. (TIF) [file pone.0220196.s002.tif]

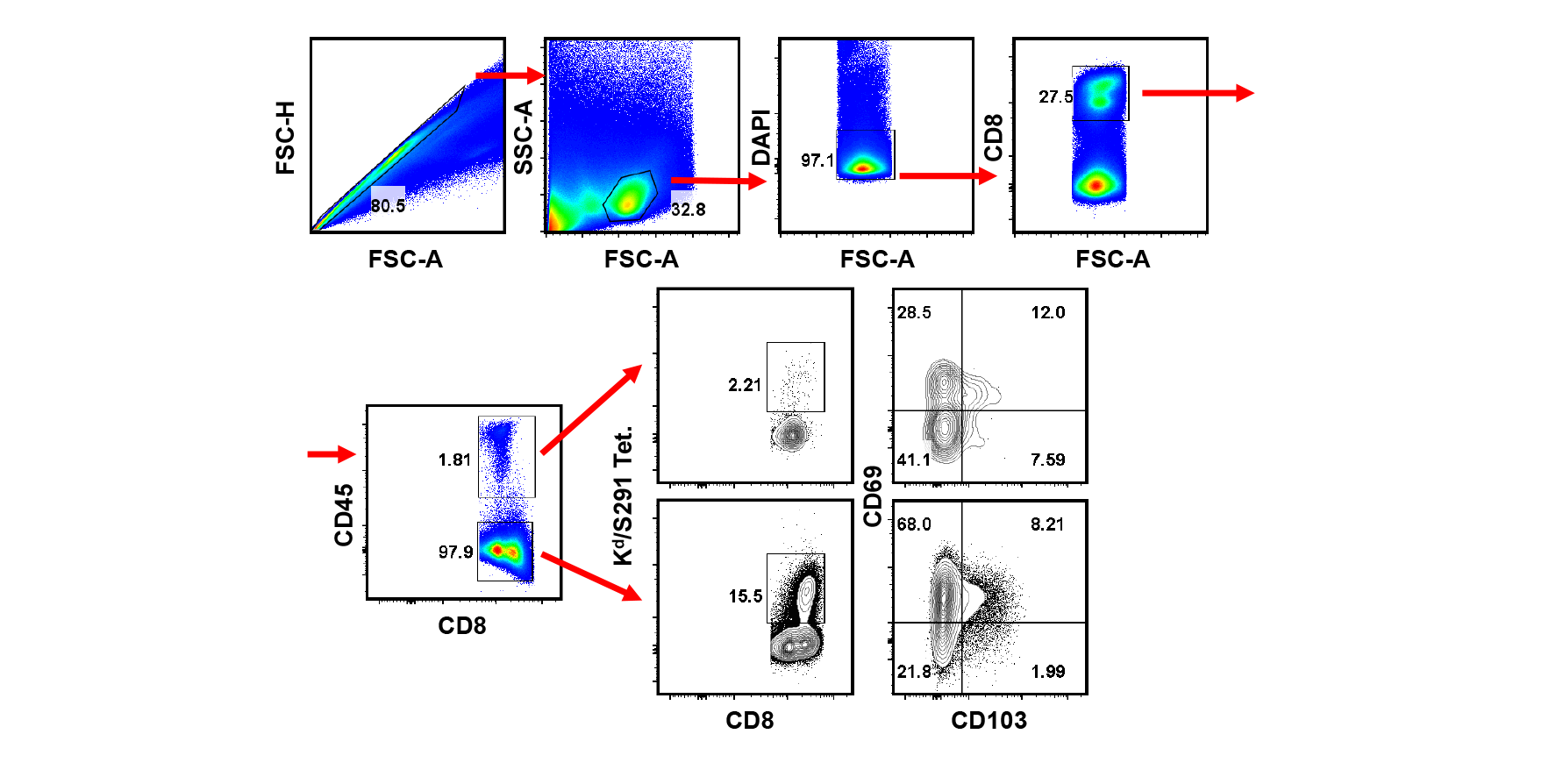

Supplement: S3 Fig — BALB/c mice (n = 4 per group) were immunized with 1×10^8^ PFUs of rAd/Spike via the IN or IM route. On day 30, mice were injected intravascularly with APC-conjugated anti-CD45 antibody and sacrificed after 5 min. BAL as well as the lungs and spleen were harvested and cells were stained with Kd/S291 tetramer, DAPI, anti-CD8, anti-CD103, and anti-CD69 antibodies. Cells were gated for singlets (FSC-A/FSC-H), lymphocytes (FSC-A/SSC-A), and live cells (FSC-A/DAPI−). Then, lung cells were discriminated as follows: lung parenchyma cells (Kd/S291 Tet+CD8+CD45–) and lung vasculature cells (Kd/S291 Tet+CD8+CD45+). (TIF) [file pone.0220196.s003.tif]
